# Editorial for Special Issue “Cellular and Molecular Biology Insights into Neurodegenerative Diseases: From Pathogenesis to Therapeutic Targets”

**DOI:** 10.3390/cimb48010044

**Published:** 2025-12-29

**Authors:** Michela Pecoraro, Maria Pascale, Silvia Franceschelli

**Affiliations:** Department of Pharmacy, University of Salerno, Via Giovanni Paolo II, Fisciano, 84084 Salerno, Italy

## 1. Introduction

The field of neurodegenerative diseases represents one of the most challenging and fascinating areas of modern cellular and molecular biology. Diseases such as Alzheimer’s, Parkinson’s, spinocerebellar ataxias, and other related disorders are characterized by progressive neuronal loss, often associated with the accumulation of misfolded proteins, cellular stress, chronic inflammation, and metabolic alterations [1,2,3,4]. This Special Issue of the journal *Current Issues in Molecular Biology*, entitled “Cellular and Molecular Biology Insights into Neurodegenerative Diseases: From Pathogenesis to Therapeutic Targets”, offers an up-to-date, multidisciplinary overview of these topics, bringing together original research, preclinical model studies, and bioinformatic analyses. In this editorial, we want to reflect on the broader landscape of neurodegenerative diseases, emphasizing how the contributions in the Special Issue are part of an ever-evolving research trajectory.

## 2. Relevance of Neurodegenerative Diseases

In recent decades, the aging of global populations has made neurodegenerative diseases a major public health priority. Alzheimer’s, Parkinson’s, and hereditary forms such as spinocerebellar ataxia type 7 (SCA7) not only cause enormous human and social impact but also represent extraordinary natural laboratories for studying nerve cell biology, protein degradation, oxidative stress, and the interaction between the central and peripheral nervous systems.

Underlying many of these diseases are shared mechanisms such as impaired proteostasis, endoplasmic reticulum (ER) stress, mitochondrial dysfunction, and inflammation, as well as alterations in microRNA and regulatory networks. However, each disease also has distinct features: for example, Alzheimer’s is dominated by the accumulation of β-amyloid and Parkinson’s by the loss of dopaminergic neurons, while in SCA7, the genetic cause is the expansion of CAG triplets. This complexity requires equally varied and integrated approaches, such as those included in this collection of articles.

By analyzing the seven articles published in the Special Issue, four major areas of research can be identified:

- Biomarkers and early diagnosis;
- Molecular mechanisms of pathogenesis;
- Therapeutic strategies based on natural or peptide compounds;
- Gene regulation via microRNA.

These areas reflect current trends in the world of neurodegenerative biology: the need to identify early signs of disease, understand how cells become dysfunctional, and finally find molecular interventions that can modify the course of the disease.

## 3. Overview of Key Contributions

Below, we focus on some of the most representative contributions to this Special Issue to illustrate how each fits into the broader framework of research on neurodegenerative disease.

### 3.1. Plasma Biomarkers of Aβ

The study “Amyloid Beta as a Candidate Blood Biomarker of Early Cognitive Decline in the Elderly” by McFarlane et al. [contribution 1] explores the relationship between plasma concentrations of β-amyloid and cognitive status in older adults. Although it found no significant differences between groups (control, mild cognitive impairment, mild dementia), this work is crucial because it highlights the challenges in identifying non-invasive biomarkers of Alzheimer’s disease and the urgency of larger prospective studies to validate such potential indicators.

### 3.2. Peripheral Immune Modulation and Alzheimer’s Disease

The article by Klein, Gofrit, and Greenblatt, “BCG Impact on PD-1/PD-L1 Expression in Peripheral Immunocytes…” [contribution 2], presents retrospective results on patients treated with BCG for non-muscle-invasive bladder cancer, showing changes in PD-1 and PD-L1 expression in peripheral blood mononuclear cells. These data suggest a potential link between peripheral immune checkpoint modulation and protection against Alzheimer’s disease, opening an unexpected therapeutic perspective: not only the brain, but also the peripheral immune system may be a target for preventive or therapeutic strategies.

### 3.3. Natural Neuroprotective Compounds

In “Evaluation of the anti-Alzheimer’s activity of *Lycium barbarum* polysaccharide…” [contribution 3], Lu et al. analyze the effect of polysaccharide derived from Goji (*Lycium barbarum*) in an animal model of Alzheimer’s induced by Aβ_1–42_. The results show that LBP improves cognitive function and positively modulates both inflammatory pathways (e.g., TNF-α, IL-1β, IL-6) and oxidative stress, suggesting a multifactorial and potentially safe action as an adjunct in therapeutic strategies.

In the broader context of dementia, the review by Szala-Rycaj and colleagues, “Neuroprotective potential of phytochemicals…” [contribution 4], summarizes evidence on polyphenols, alkaloids, and terpenoids in animal models of (scopolamine-induced) Alzheimer’s disease. The authors point out that many of these compounds show neuroprotective effects while maintaining low toxicity and could thus represent viable alternatives or adjuncts to existing drugs. Despite this, challenges related to bioavailability and clinical translation remain high.

### 3.4. Protein Aggregation-Inhibiting Peptides

The study “Molecular Integrative Study on Inhibitory Effects of Pentapeptides on Polymerization and Cell Toxicity of Amyloid-β Peptide (1–42)” by Ye et al. combines computational docking, molecular dynamics, and experimental testing to identify pentapeptides (specifically TRRRR and ARRGR) capable of binding to Aβ_42_, inhibiting its aggregation, and reducing cellular toxicity (ROS, apoptosis) in SH-SY5Y cells. This integrated approach represents a paradigm of how bioinformatics and experimental biology can converge to design innovative therapeutic molecules [5].

### 3.5. Genetic Diseases: microRNA and SCA7

The article “In Silico Analysis of miRNA-Regulated Pathways in Spinocerebellar Ataxia Type 7” by Borgonio-Cuadra et al. uses bioinformatic analysis to identify targets of four microRNAs (miR-29a-3p, miR-132-3p, miR-25-3p, and miR-92a-3p) implicated in the pathogenesis of SCA7. The authors highlight key pathways: adherens junctions, neurotrophic signaling, ER processing, cytoskeleton, apoptosis, and dopaminergic synapses. This study not only illuminates the regulatory complexity of SCA7 but also suggests potential microRNAs that could be targeted for therapy [6].

### 3.6. Preclinical Models of Parkinson’s Disease and Natural Therapy

In the article “Effects of Mucuna pruriens (L.) DC. and Levodopa in Improving Parkinson’s Disease in Rotenone Intoxicated Mice,” Zaigham and Paeng show that an extract of Mucuna pruriens, rich in L-DOPA, has effects comparable to synthetic L-DOPA in a rotenone-induced mouse model of Parkinson’s disease. Treatment with MP (Mucuna pruriens) reduces peripheral inflammation (IL-6, TGF-β1) and improves motor and olfactory performance. This ancient therapeutic strategy is becoming a modern alternative that is potentially more sustainable and economically accessible [7].

## 4. Prospects

The articles included in this Special Issue highlight some strategic points and future developments in the field of neurodegenerative diseases:

- Systemic approaches: It is not enough to study the brain in isolation. The article on BCG and peripheral immune checkpoints indicates that immune modulation outside the central nervous system can influence the risk or progression of disease. This opens up new and more accessible therapeutic scenarios.
- Multi-target/natural therapies: The works on *Lycium barbarum* and *Mucuna pruriens* shows how natural compounds can act on multiple mechanisms—inflammation, oxidative stress, proteostasis—and offer less invasive or more complementary clinical modalities than conventional treatments.
- Precisely designed molecules: The study on pentapeptides represents the cutting edge of molecular chemistry. Through computational techniques, we can design small molecules capable of preventing the toxic aggregation of pathological proteins, an approach that could eventually translate into therapies with fewer side effects than monoclonal antibodies or other large molecules.
- Gene regulation and microRNAs: The pathogenesis of hereditary diseases such as SCA7 cannot be understood by analyzing the mutated gene alone. MicroRNAs and the regulatory networks they control represent an additional dimension that offers potential innovative therapeutic targets.
- Biomarkers and early diagnosis: The failure to find a significant difference in plasma Aβ levels in McFarlane et al.’s sample highlights one of the biggest challenges in neurodegenerative research: that of how to detect the disease in its early stages, before neuronal damage becomes irreversible. This will require longitudinal studies, more sensitive techniques, and perhaps combinations of biomarkers (plasma, immune, and genetic).

## 5. Conclusions

Neurodegenerative diseases remain a biological and clinical enigma of extraordinary complexity, but also a powerful driver of multidisciplinary innovation. This Special Issue of *Current Issues in Molecular Biology* demonstrates how contemporary research is embracing systemic approaches, combining bioinformatics, animal models, natural compounds, and molecular design, not only to discover the pathogenesis of these diseases but also to translate these discoveries into potential therapeutic strategies.

From an editorial perspective, we hope that this collection will stimulate new collaborations between molecular biologists, immunologists, medicinal chemists, bioinformaticians, and clinicians. Many challenges remain: improving the sensitivity and specificity of biomarkers, optimizing the bioavailability of natural or peptide compounds, and ensuring that emerging therapies are also accessible and sustainable. At the same time, the outlook presented by these contributions is optimistic: cellular and molecular biology has the tools to radically transform the way we understand and treat neurodegenerative diseases.

We would like to thank the authors who contributed to this Special Issue, the reviewers for their rigorous comments, and the readers for their interest and commitment to the field. The challenge of neurodegeneration is not only scientific, but also human, and requires an integrated vision of research, care, and hope.
